# Targeting microRNAs: a new action mechanism of natural compounds

**DOI:** 10.18632/oncotarget.14392

**Published:** 2016-12-30

**Authors:** Qian Lin, Leina Ma, Zhantao Liu, Zhihong Yang, Jin Wang, Jia Liu, Guohui Jiang

**Affiliations:** ^1^ College of Medicine, Qingdao University, Qingdao, China; ^2^ The Department of Oncology, The First Affiliated Hospital of Qingdao University, Qingdao University, Qingdao, China

**Keywords:** natural compounds, miRNAs, cancer

## Abstract

Unlike genetics, epigenetics involves the modification of genome without changes in DNA sequences, including DNA methylation, histone modification, chromatin remodeling and noncoding RNA regulation. MicroRNA (miRNA), a member of noncoding RNAs superfamily, participates in RNA interference through a unique mechanism. Currently, microRNAs have been found to be regulated by some natural compounds. Through altering the expression of miRNAs and influencing the downstream signaling pathways or target genes, several natural compounds exhibit its bioactivity in the prevention, diagnosis, therapy, prognosis and drug resistance of human diseases, such as cancer. In this review, several natural compounds and their studies about miRNA-related action mechanism were summarized. These studies provide a new insight into action mechanism by which natural compound exerts its bioactivity and a novel treatment strategy, demonstrating natural compound a promising remedy for clinical treatments.

## INTRODUCTION

**Figure 1:**
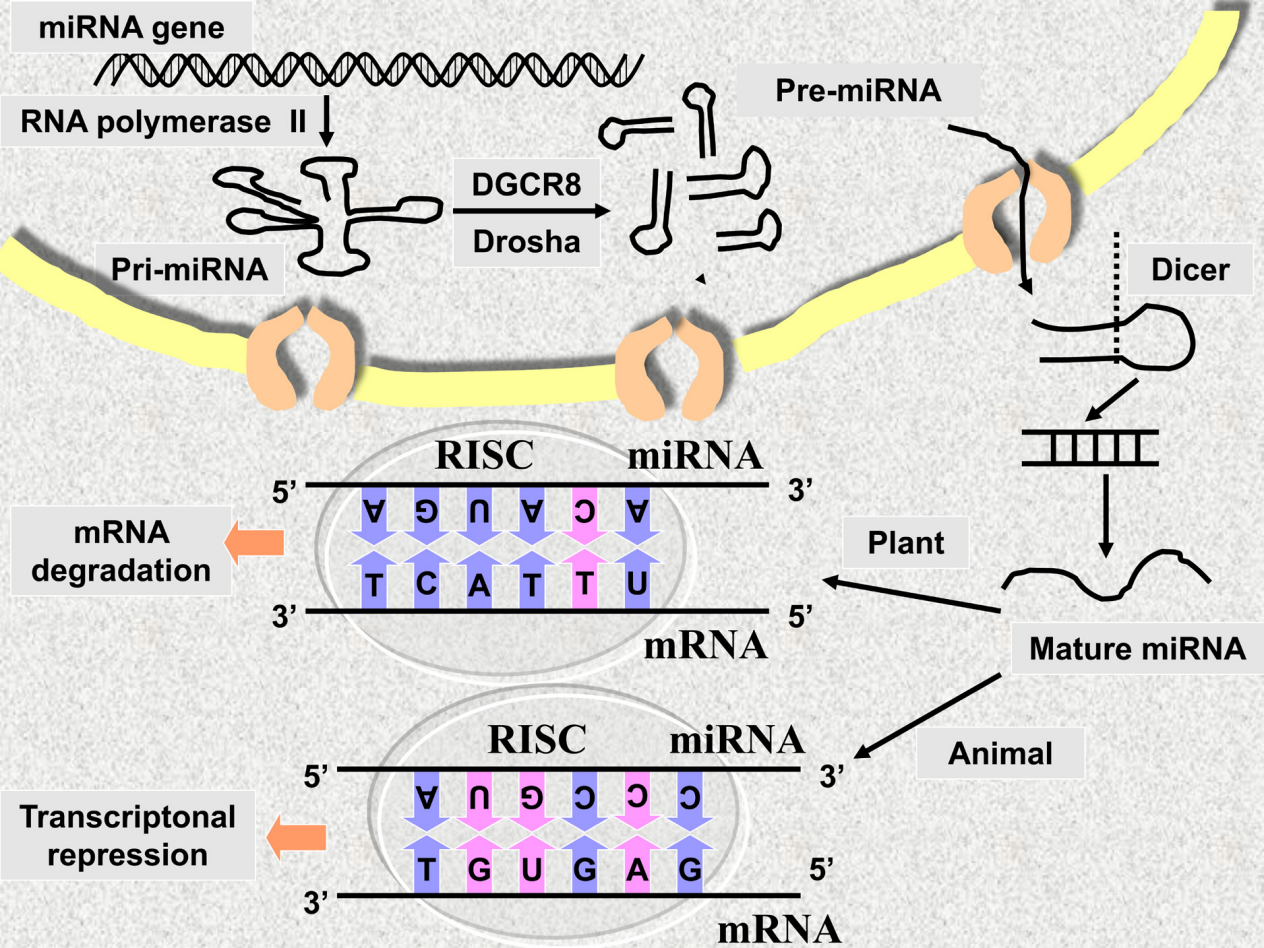
The mechanism of miRNA action

MicroRNA (miRNA), a kind of non-coding RNAs, which is generally delineated as endogenous around 22 nt RNAs that can play important regulatory roles in animals and plants by targeting mRNAs for cleavage or translational suppression [1]. In animals, post-transcriptionally, miRNA binds to the 3’ untranslated region (3’-UTR) of target mRNA *via* imperfect matching, which results in inhibition of translation. In plants, miRNA leads to mRNA degradation through perfect or nearly perfect matching with the open reading frame (ORF) of target mRNA (Figure 1). It has been reported that conserved miRNA targets cover more than one third of human genes [2]. Numerous studies have revealed miRNAs’ function in cancer development and its aberrant expression profiles compared to normal cells in human malignancy. Moreover, miRNAs even involves in action mechanism of antitumor drugs, at least in part. All these features make miRNA a promising biomarker [3] and drug target for diagnose, therapy and prognosis.

Natural compounds and its derivatives have been widely used into clinical treatment due to its innovative structure, high biological activity and low side effects. However, its complicated mechanism is difficult to be elucidated, which limit its application. Increasing evidence has revealed that some natural compounds exhibit anticancer properties through epigenetic regulation [4]. During its antitumor action, natural compounds have been shown to alter expression profiles of certain miRNAs by unknown mechanisms. In this review, these miRNA-associated natural compounds are listed as examples to describe this phenomenon and summarized for their possible action mechanisms.

## NATURAL COMPOUNDS AFFECTING MIRNAS

### Docosahexaenoic acid (DHA)

Docosahexaenoic acid (DHA) is a kind of unsaturated fatty acid that exists in fish, seaweed oil and egg yolk, etc., which is a common ingredient in the infant formula for its vital importance in the development of baby intelligence and vision. In current investigation, DHA works as a preventative drug and an adjuvant drug to breast cancer treatment with unclear mechanisms. One of reported anticancer mechanisms of DHA is suppression of tumor angiogenesis. Recently, microRNAs in secreted exosomes, which can be transferred to endothelial cells where they induce pro-angiogenic effects encourages a research group to investigate the relationship between DHA and exosomal miRNAs. Results revealed that DHA both increases exosome secretion and miRNAs (let-7a, miR-21, miR-23b, miR-27b, and miR-320b) levels in exosomes and these miRNAs are also increased in the endothelial cells by DHA treatment of MCF7 cells. This study indicates that, in breast cancer, DHA is likely to inhibit angiogenesis through stimulating exosome secretion and altering exosomal miRNAs [5].

### Resveratrol

Resveratrol is a polyphenolic phytoalexin abstracted from peanut, mulberr and red wine, etc., which has various bioactivities, such as anticancer, immunoregulation and protection against cardiovascular disease [6]. Studies have reported that resveratrol suppresses glioma cell growth by down-regulating oncogenic microRNAs (miR-21, miR-30a-5p, miR-19) and up-regulating their targets’ expression, which are closely relevant to glioma formation and development [7]. Resveratrol has also been found to decrease the portion of breast cancer stem-like cells by increasing Argonaute2 (Ago2, a component of RNA-inducing silencing complex, RISC) expression through enhancing promoter activity and prolonging half lives of mRNA and protein. In this process, many tumor-suppressive miRNAs are also increased. Similarly, dimethylether resveratrol analogue pterostilbene has also been shown to exert antitumor activity in a miRNA-dependent manner. This finding indicates that this activity is likely common in stilbene family containing resveratrol [8].

### Curcumin

Curcumin is a powerful polyphenolic and antioxidant compound found in turmeric. Preclinical studies suggested that curcumin shows an antitumor effect on diverse carcinomas, including retinoblastoma, pancreatic cancer, glioma, lung cancer, colon cancer, hepatoma, breast cancer, cervical carcinoma, leukemia, gastric carcinoma, prostate cancer, melanoma, oral epithelial cancer, bladder cancer and ovarian cancer, etc. [9]. Recent studies have summarized the epigenetic alteration induced by curcumin [10]. Many investigations have been focused on miRNA to demonstrate curcumin's action mechanism. In pancreatic cancer cells, curcumin inhibits cell growth and invasion through up-regulation of miR-7 and down-regulation of its oncogenic target, lysine methyltransferase SET-8 [11]. MiR-146a, a negative regulator of nuclear factor-kappaB (NF-κB) signaling that promotes tumor growth and survival, is up-regulated in the curcumin-mediated sensitization of human glioblastoma cells to temozolomide [12]. A tumor-suppressive miRNA, miRNA-203, is frequently downregulated in bladder cancer. Curcumin has been found to promote its expression and block its target genes Akt2 and Src, ultimately inducing apoptosis and inhibiting proliferation [13]. In cisplatin-resistant human lung carcinoma cells A549/DDP, curcumin inhibits the overexpression of miR-186*, leading to elevated apoptosis and declining survival rate with unclear action mechanism [14]. In chronic myelogenous leukemia cells, the reduction of miR-21 in exosomes may be related to the antileukemic activity of curcumin [15].

### (6)-Gingerol (6G)

(6)-Gingerol is a natural polyphenolic alkanone with antitumor and anti-inflammatory properties. Previous studies have reported that miRNA is also associated with the antitumor activity of 6G. By means of suppressing mitochondrial respiratory complex (MRC I), 6G induced the generation of reactive oxygen species (ROS) followed by DNA damage and up-regulation of miR-27b, and in turn, miR-27b inhibits the PPARy-NF-κB pathway and leads to the apoptosis of myeloid leukemia cells [16].

### Diaporine A

Diaporine A is a bioactive substance isolated from endophytic fungus 3lp-10. A study has demonstrated that diaporine A can inhibit non-small cell lung cancer growth by up-regulating miR-99a, which is followed by the suppression of mTOR signaling pathway to achieve its antitumor effects [17].

### Berberine and Berbamine (BBM) derivate BBMD3

Berberine is a frequently-used alkaloid which possesses a significant anti-inflammatory effect. It is broadly used against infective disease of alimentary canal clinically. Current analysis has revealed that in multiple myeloma cells, berberine can promote the expression of programmed cell death 4(PDCD4) *via* the restoration of miR-21, an oncomir that involves in tumor protein p53 (TP53) signaling and other cancer pathways [18].

BBMD3, a berbamine (BBM) derivate, is found to inhibit cell viability and induce apoptosis in cancer stem-like cells (CSCs) of human glioblastoma. The up-regulation of miR-4284 can partially accounts for antitumor mechanism of BBMD3, although the relevant signaling cascade is not completely clear [19]. CXCL5, which has been reported to function for various cancer development, has been confirmed to be a direct target of miR-4284 [20], suggesting that CXCL5 might be also involved in BBMD3′s effect. .

### Aplysin

Aplysin is a marine brominate compound that can not only suppress malignancy, but also eliminate drug-resisitance of cancer cells. Since miR-181b-abundant glioma cells is more sensitive to temozolomide (TMZ) [21], aplysin enhances TMZ action through increasing the expression of miR-181 in TMZ-resistant glioma cells, which results in MEK1 (mitogen-activated protein kinase kinase 1) downregulation [22].

### Methyl jasmonate

Drug combination is a novel strategy for clinical medication for its great synergy, such as methyl jasmonate and gambogic acid (GA). Studies have revealed that the sensitivity of GA-treated human bladder cancer cells to apoptosis triggered by methyl jasmonate is relevant to the down-regulation of enhancer of zeste homologue 2 (EZH2) level by the elevation in the expression of miR-101, which directly targets EZH2. Their combination increases the antitumor efficacy compared to GA alone [23].

### Ellagitannin (BJA3121) and BJA32515

Ellagitannin (1,3-Di-O-galloyl-4,6-(s)-HHDP-b-D-glucopyranose, BJA3121) and BJA32515 (1,3,4-tri-O-galloyl-6-O-caffeoyl-beta-D-glucopyranose) are polyphenolic compounds extracted from Balanophora Japonica Makino and exhibit antiproliferative effect. Giving the fact that miRNAs play critical roles in cancer cell proliferation, studies have demonstrated that ellagitannin BJA3121 and BJA32515 are likely to inhibit HepG-2 cancer xenograft growth by modifying miRNA expression profiles [24, 25].

### Genistein

Genistein is an isoflavonoid isolated from dietary soybean, it has been found to inhibit several oncomirs’ expression and promote tumor-suppressing miRNAs’ expression to function as a promising antitumor drug. In pancreatic cancer cells, genistein inhibits cell proliferation and increases apoptosis by down-regulating miR-223 [26] and up-regulating miR-34a expression [27], whose targets Fbw7 and Notch-1 are upregulated and downregulated, respectively. In ovarian cancer cells, genistein inhibits cell migration and growth *via* reducing overexpressed miR-27a accompanied by increasing its target, Sprouty2 [28]. In prostate cancer cells, genistein inhibits invasion and migration by its down-regulation of miR-151 [29] and up-regulation of miR-574-3p [30].

### 1’S-1’-acetoxychavicol acetate (ACA)

1’S-1’-acetoxychavicol acetate (ACA) is a natural product and reported to serve as a chemosensitizer. ACA can potentiate the antitumor activity of cisplatin on human cervical cancer cell line, Ca Ski by altering miRNAs that are predicted to target genes associated with cell cycle progression and apoptosis [31]. The changes in miRNA level between the administrations of cisplatin alone and cisplatin plus ACA are remarkable.

### 10’ (Z), 13’ (E), 15’ (E)-heptadecatrienylhydroquinone [HQ17 (3)]

10’ (Z), 13’ (E), 15’ (E)-heptadecatrienylhydroquinone [HQ17 (3)] is a natural compound purified from the sap of the lacquer tree Rhus succedanea with antileukemia activity. Studies on its pharmacological mechanism indicated that HQ17 (3) down-regulates miR-17-92 cluster expression *via* decreasing one of their upstream regulators c-Myc, which might be involved in its inhibitory effect on leukemia cells [32].

### 3, 6-dihydroxyflavone (3, 6-DHF)

Considering that some miRNAs are relevant to tumorigenesis and development, 3,6-DHF was employed to study *in vivo* and *in vitro* to investigate whether some miRNAs play roles in the antitumor effect of flavonoids. It is found that 3,6-DHF activates apoptosis in breast cancer cells *via* reversing the upregulation of miR-21 and the down-regulation of miR-34a induced by tumorigenic 1-methyl-1-nitrosourea (MNU), indicating that miR-34a and miR-21 are partly relevant to flavonoids’ inhibition of tumorigenesis and promotion of apoptosis [33].

### Andrographolide

Andrographolide is abstracted from *Andrographis paniculata*, which is famous for its anti-inflammatory activity. Accumulated studies have been reported its inhibition of hepatoma tumor growth *in vivo* and *in vitro*. Recent studies found that andrographolide can upregulate the expression of some miRNAs, including miR-222-3p, miR-23a-3p, miR-106b-5p and miR-30b-5p. Target genes of these miRNAs are also downregulated. All of them are likely to be related with the anti-hepatoma activity of andrographolide [34].

### Prodigiosene

Prodigiosene (methyl-3-pentyl-6-methoxyprodigiosene) is a kind of secondary metabolite abstracted from the cell wall of *Serratia marcescens.* A latest Iran study found that prodigiosene can induce apoptosis in colorectal cancer stem-like cells by increasing the expression of miR-16-1 and decreasing its target gene, survivin. As it is believed that overexpression of survivin in cancer stem-like cells lead to the failure of clinical drug administration, prodigiosebne could be a good choice for fighting against this obstacle in counties with high morbidity and mortality of colorectal cancer, such as Iran [35].

### Oleanolic acid

Oleanolic acid is common in the ingredient of ladies’ make-up. Moreover, it is also a natural anti-carcinoma compound. A current study has demonstrated that oleanolic acid exerting its antitumor activity by upregulating miR-122, a tumor suppressor in lung cancer *in vivo* and *in vitro*. In addition, overexpression of CCNG1 (cyclin G1) and MEF2D (myocyte enhancer factor 2D), two targets of miR-122 also abolished the antitumor effect in lung cancer, which indicates that oleanolic acid may inhibit lung cancer growth through miR-122/CCNG1/MEF2D pathway [36].

## IDENTIFICATION OF NATURAL COMPOUND-RELATED MIRNAS

Taken together, there are some remarkable regulations in the identification of natural compound-associated miRNAs. (1) Bioactivities of some miRNAs are similar with the pharmacological effects of a given natural compounds on cancer cells [5, 7, 8, 16,19, 21, 22, 24–27, 29, 30, 31], this similarity encourages researchers to wonder whether natural compound exhibits its effect by affecting these miRNAs. For example, miR-181 is related to TMZ-resistance of glioma, and interestingly, aplysin can also enhance the sensitivity of glioma cells to TMZ. Thus, researchers investigated their association and found aplysin exerts its function through increasing the expression of miR-181. (2) If the target of a natural compound is identical to the potential target of a certain miRNA, it do deserves further investigation about their relation [11, 12, 15, 17, 18, 23 ,32]. For example, methyl jasmonate and miR-101 share a common target, EZH2. Studies have been carried out to investigate their relation and found that methyl jasmonate down-regulated EZH2 level by the elevation in the expression of miR-101. (3) Some miRNAs play roles in the formation and progression of cancer and aberrantly expressed in cancer cells compared with normal counterpart. If results from qRT-PCR and microarray analysis indicate that a certain natural compound can reverse the aberrant expression profiles of some miRNAs, it is likely that natural compound exerts its effect through altering these miRNA expression profiles [13, 14, 21, 28, 33]. For example, the expression of a tumor-suppressive miRNA, miR-203 is rather low in bladder cancer. Nevertheless, curcumin increases miR-203′s expression and then inhibits cancer cell growth to achieve its antitumor activity.

## CONFIRMATION OF MIRNAS AS A NATURAL COMPOUND'S TARGETS

Confirming a miRNA candidate as an authentic target of natural compounds includes the following steps: confirmation of miRNA candidates, discovery of the mechanism by which a natural compound influences these miRNA candidates, determination of the roles that miRNA plays in natural compound's antitumor effect, exploration of the action mechanism of miRNA-mediated antitumor effect of natural compound, and at last, *in vivo* evidence from animal experiments.

### Confirmation of miRNA candidates

Usually, microarray analysis can be carried out to determine miRNAs with altered expression level after the treatment of a natural compound. The miRNA candidates that are overexpressed or underexpressed by more than two folds are selected for further study to confine the measurement error. However, the chosen miRNAs are still needed to be verified by qRT-PCR.

### Discovery of the mechanism by which a natural compound influences these miRNA candidates

Generally, gene regulation is so precise that cells can employ them to adapt to the changing complicated environments. As a non-coding gene, the regulation of miRNA is also believed to accurately response to the treatment of natural compounds, by diverse delicate mechanisms. The possible mechanisms are listed as below:

Impact on the transcription of primary miRNA

Primary miRNA transcript is the first step of miRNA biogenesis (Figure 1). Transcriptional factors related to this process may mediate the influence on the level of miRNA by natural compounds. Transcriptional factors modulating miRNAs could be collected from TransmiR database [37].

In addition, miRNA expression has also been reported to be affected by epigenetic regulation such as histone modification and DNA methylation [38]. Hypermethylation in the CpG islands of miRNA's promoter is responsible for its epigenetic silence in several human cancers [39].

A usual epigenetic agent that could inhibit DNA methyltransferase in cancer cells, 5-aza-2’-deoxycytidine (5-Aza-dC), exhibit maximal DNA de-methylation activity without killing cells at a suitable concentration [40], and thus, 5-Aza-dC can be applied to test whether the methylation of miRNA's promoter is responsible for its dysregulation. In non-Hodgkin's lymphoma and multiple myeloma, miR-129-2 serves as a tumor suppressor and its promoter is frequently methylated [41]. In gastric cancer, miR-196b acts as an oncomir and it is overexpressed because of hypomethylation of its CpG islands [42]. In prostate cancer, miR-31 plays a role as an antioncogene and its promoter is hypermethylated [43]. Similarly, miR-203 is a tumor suppressor and it is inactive due to the hypermethylation of its promoter in bladder cancer cells. However, miR-203 expression can be augmented by curcumin with partial demethylation of its promoter [13].

(2) Impact on miRNA processing and maturation

The post-transcription process of miRNA biogenesis is described as follow: Primary miRNA is cleaved into precursor miRNA, and in turn, these precursors are transferred from the nucleus into cytoplasm (Note: primary and precursor miRNA are both hairpin RNAs with double strands). Subsequently, precursor miRNAs are processed by Dicer into shorter fragment without hairpin segment. This shorter fragment contains mature miRNA and its complementary strand. The useless complementary strand will be discarded at last (Figure 1). In these steps, certain RNA-binding proteins [44] participate can be the targets of natural compound.

Aberrant processing of mature miRNA could be evaluated by the ratio of mature miRNA and precursor miRNA (M/P ratios), which can be calculated based on the abundance of mature miRNA and precursor miRNA measured by deep sequencing analysis. In human glioma, the M/P ratios of many miRNAs are higher than normal brain, and are increased along with the severity of glioma. Moreover, systematical abnormal expression of genes relevant to miRNA processing in nucleus, transport from nucleus to cytoplasm, and processing in cytoplasm takes place in glioma tissues [45].

It has been reported that some anticancer chemicals exhibit affinity binding to the pre-miRNA of miR-155 to prevent its cleavage from Dicer, and therefore, interfere with mature miR-155 synthesis [46].

(3) Indirect mechanisms

Indirect mechanisms are unrelated to miRNA biogenesis, such as miRNA-sponging effect of circular RNA [47] and long non-coding RNA (lncRNA) [44], which may also explain the modulation of natural compounds on miRNA expression.

It should be highlighted that regulation of different miRNAs modulated by one natural compound can be precise and specific. For example, in human pancreatic cancer cells treated with circumin, miR-181d is significantly changed, but miR-181c (chr19:13874699-13874808), which is close to miR-181d (chr19:13874875-13875011) in the genome, is not changed [48].

### Determination of the roles that miRNA plays in natural compound's antitumor effect

Subsequently, it is necessary to evaluate if the interference of miRNA level by transfection of precursor mimics or antagomir antisense could rescue the effects of the natural compounds, such as cell cycle arrest, cytotoxicity, anti-migration and anti-invasion activities. If this interfering can reverse the effect of natural compounds, it can be concluded that the natural compound might exert its effect *via* certain miRNAs.

### Exploration of the action mechanism of miRNA-mediated antitumor effect of natural compounds

To further investigate which target or downstream signaling pathway of miRNA ultimately accounts for the dependence of natural compound's antitumor activity on miRNA, some studies have been conducted to figure out more precise molecular mechanisms.

Differential gene expression profiles before and after the treatment of the natural compound assessed by proteomic technology [49] or CRISPR-Cas9 genome editing technology [50] could be taken together with the target genes of miRNAs predicted by several software [51], such as DIANA-microT 3.0 [52], RNA22, miRDB, miRanda, Tarbase [53] and TargetScan to find the overlapped genes, that is, the *bona fide* target genes that mediate the miRNA-relayed effect of natural compounds. In addition, target genes collected from the overlapped data from at least thee target prediction software of miRNAs could also be selected as candidates for later analysis. Both of the results above need to be filtered and confirmed by qRT-PCR.

Then, signaling pathways associated with the target genes could be enriched by Kyoto Encyclopedia of Genes and Genomes (KEGG). Gene Ontology (GO, http://www.geneontology.org) is an international gene function system, the distribution of gene function in GO analysis could be studied by KEGG to elucidate the appearance of gene function due to the difference of sample. By employing pathway significance enrichment analysis, target genes-related signaling pathways with remarkable enrichment contrast to the whole genome could be indentified easily.

In this regard, the network of a natural compound-miRNAs-target genes could be printed [34].

Besides, Whether a natural compound could effect on the downstream signaling pathway of miRNA or not can be tested by building up luciferase reporting vector containing promoter of downstream elements’ genes and detecting the fluorescence intensity. And with the help of siRNA expression cassettes (RNA interference) and overexpression vector ( such as pcDNA and Dox ,) , miRNA target genes’ expression can be altered to investigate if the natural compound could also take into effect.

It must be noted that the regulation of a gene is a combinational and complex result, the degradation from miRNA may not be the main point, influenced activity of the gene promoter and the cleavage of the gene translated protein by some enzyme, such as caspase3, could also make sense. This phenomenon is reflected on the distinct effect on miRNAs under different concentration of a natural compound [54]. Moreover, it is believed that there are negative feedback regulation between miRNA and its targets. For example, downregulation of miR-34a/b/c increase the expression of its target, SNAIL. In turn, SNAIL suppresses the miR-34a/b/c expression by binding to the E-boxes in the miR-34a/b/c promoters [55].

### Acquiring *in vivo* evidence from animal experiments

Moreover, animal experiments are also necessary to obtain *in vivo* evidence. Xenagroft or orthotopic transplantation tumor model are established by intraperitoneal injection, intravenous injection or subcutaneous injection, followed by periodical administration referencing clinical experience of natural compounds at proper doses or maximum tolerated dose (MTD). Then, some fundamental measurement should be performed in virtue of *in vivo* imaging system (IVIS) [56], such as tumor size, weight, latency, and incidence. If the effect of the given compounds on the invasion and metastasis of tumors are aimed to be tested, the mice should be sacrificed and tumor metastasis must be counted.

## CONCLUSIONS AND PERSPECTIVES

Currently, most researchers focused antitumor mechanism of natural compounds on protein, but more and more evidence has revealed that miRNAs can also be related to tumor suppressor activity of natural compounds. The general action mechanism by which natural compound exerts its miRNA-dependent antitumor function is as follows: a natural compound exhibits its effects through up-regulating or down-regulating miRNAs to suppress or activate their target gene expressions or their relevant signaling pathways. Moreover, the components of RISC can also be promoted by natural compounds. Some issues are still to be addressed.

Beside microRNA, other kinds of non-coding RNA, such as lncRNA (long non-coding RNA), small interfering RNA (siRNA), piRNA (Piwi-interacting RNA) and circRNA (Circular RNA) may also be involved in the action mechanism of anticancer drugs. These RNAs are also of unexpected and remarkable effect on malignance. CircRNA is stable and conserved in cytoplasm, it acts as more than miRNA sponge. Recent study summarized that cirRNA regulates tumor-related pathways together with miRNA, linear RNA transcription and protein production [47]. LncRNA is larger than other non-coding RNAs with more than 200 nt, it plays important roles in various processes, such as chromatin looping, protein scaffolding, gene transcription, post transcriptional processing, miRNA sponge [57], endocrine resistance [58] and so on. It has been reported that genistein inhibits cell proliferation in prostate cancer cells through reduction of an oncogenic lncRNA, HOTAIR [59]. SiRNA is usually employed in gene knock-down, it acts similar posttranscriptional mechanism to miRNA. After cleaved by Dicer, long double strand RNA are teared into many siRNAs, among them, the siRNAs complementing the mRNA transcribed by the target gene will interact with RISC and then unfolded to sigle strands. The antisense will bind the target mRNA and degrade it. PiRNA defends the genome against insertional mutation caused by transposon in germ cell lines and benefits the somatic gene regulation by sequence-specific histone modification and DNAmethylation. In human breast carcinoma, piRNA is reported to make sense through remodeling the tumor epigenome [60].

**Figure 2:**
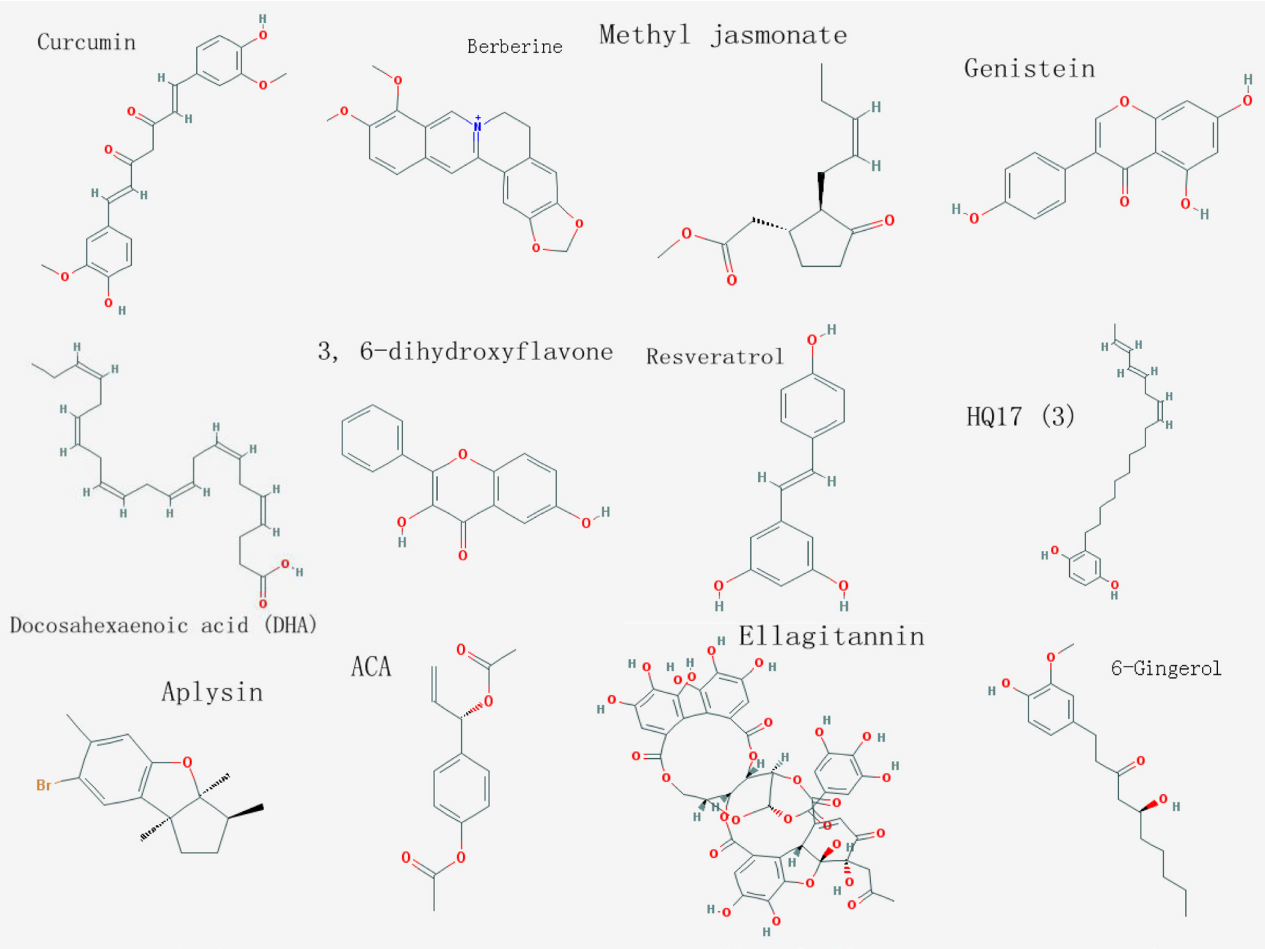
Structures of most listed natural compounds

In addition, the significance of these studies is profound, which not only serves as the new evidence for designing of novel miRNA-targeting therapeutic strategy for cancer, but also provides a new insight into our understanding on the molecular mechanism underlying human malignancy. Particularly, detecting the levels of tissue/organ-specific miRNAs will benefit diagnosis, therapy monitoring and prognosis of cancer. Intriguingly, most of the natural compounds above belong to polyphenolic compounds (Figure 2), suggesting that some structures in this group of compounds may have advantages of binding or targeting miRNA molecules over other compounds without similar structures. So is it possible to develop more potent and effective miRNA-targeting agents based on this type of chemical compounds? Furthermore, downstream proteins of compound-related miRNAs can also be selected as drug targets for further development.
